# Meta-analysis of laser-assisted periodontal therapy (2015–2025)

**DOI:** 10.1007/s10103-026-04866-9

**Published:** 2026-04-15

**Authors:** Ayşegül Sunar, Eda Sır, Çağan Taş, Enes Tekin

**Affiliations:** 1 European Vocational School, Department of Oral Health and Dental Health, Kocaeli Health and Technology University, Köseköy, Turkey; 2 Faculty of Dentistry, Kocaeli Health and Technology University, Köseköy, Turkey

**Keywords:** Laser, Periodontal treatment, Use of lasers in periodontology, Periodontology, Er: YAG laser, Nd: YAG laser

## Abstract

This systematic review and meta-analysis evaluated the adjunctive clinical efficacy of laser-assisted periodontal therapy compared with conventional scaling and root planing (SRP). The primary research question was whether laser systems provide additional improvements in clinical attachment level (CAL) and probing depth (PD) in patients with periodontitis. PubMed, Scopus, and Web of Science were searched for studies published between January 2015 and October 2025 following PRISMA 2020 guidelines. Randomized controlled trials and prospective clinical studies comparing laser-assisted periodontal therapy with SRP alone were included. Primary outcomes were changes in CAL and PD with a minimum follow-up of 3 months. Meta-analysis was performed using a random-effects model when sufficient quantitative data were available. Risk of bias was assessed using the Cochrane RoB 2.0 tool, and evidence certainty was evaluated using GRADE. Twenty-six studies were included in the qualitative synthesis, and quantitative meta-analysis was feasible only for Er: YAG lasers. The pooled mean difference in CAL gain favored Er: YAG adjunctive therapy (MD = 0.319 mm; 95% CI: −0.097 to 0.735 mm), without statistical significance. Moderate heterogeneity was observed (I² ≈ 54%). Evidence for diode lasers, Nd: YAG lasers, and antimicrobial photodynamic therapy showed modest short-term effects but lacked standardized data for quantitative pooling. Laser-assisted periodontal therapy may provide small adjunctive benefits over SRP alone. Er: YAG lasers showed the most consistent, though clinically limited, effects and should be considered supplementary rather than routine adjuncts.

## Introduction

The incorporation of laser systems into dentistry began shortly after the invention of the first functional laser by Theodore Maiman in 1960. Within a decade, researchers had recognized the potential of laser energy to enable precise and minimally invasive dental procedures. Early investigations primarily focused on cavity preparation and caries removal; however, technological limitations—such as unstable energy delivery, excessive heat generation, and high costs—restricted their widespread clinical adoption [1].

Over the subsequent decades, substantial technological advances led to the development of laser systems specifically tailored for dental applications, including CO₂, Nd: YAG, Er: YAG, and diode lasers, each characterized by distinct wavelengths and tissue interactions. In particular, the Er: YAG laser emerged as a cornerstone for hard-tissue applications due to its strong absorption in water and hydroxyapatite, which allows effective ablation with minimal collateral thermal damage. Refinements in pulse modulation and wavelength precision further enhanced the selectivity of laser–tissue interactions, reducing injury to adjacent structures and improving patient comfort [2–5].

During the most recent decade (2015–2025), there has been a marked shift toward evidence-based integration of lasers into periodontal therapy, supported by digital imaging, improved ergonomics, and more cost-effective portable units. In parallel, the establishment of standardized clinical protocols and advanced practitioner training has strengthened both the safety and reproducibility of clinical outcomes. Collectively, these developments have positioned laser-assisted periodontal therapy (LAPT) as a central and continuously evolving component of contemporary periodontal care [4, 6, 7].

### Laser systems utilized in periodontal therapy

Periodontal applications of laser technology predominantly involve diode, Nd: YAG, and erbium-family lasers (Er: YAG, Er, Cr: YSGG). Each system exhibits distinct photophysical properties that determine its therapeutic indications, tissue penetration depth, and bactericidal efficacy [5–7].

#### Diode lasers

Diode lasers, operating within the 810–1064 nm wavelength range, exert their effects primarily through photothermal absorption by chromophores such as melanin and hemoglobin. This property makes them particularly suitable for soft-tissue procedures, including gingival contouring, sulcular debridement, and periodontal pocket disinfection [8].

Their pronounced hemostatic effect and bactericidal capacity contribute to reduced intraoperative bleeding and improved postoperative healing. Multiple clinical trials conducted between 2015 and 2025 have reported enhanced bacterial load reduction and increased patient comfort when diode lasers are used as an adjunct to scaling and root planing (SRP). In addition, their compact design and relative affordability have made diode lasers one of the most frequently implemented systems in periodontal practice [7–11].

#### Nd: YAG lasers

The Nd: YAG laser, emitting at 1064 nm, demonstrates a strong affinity for pigmented tissues and bacterial components, enabling deeper penetration and effective debridement of periodontal pockets. Its bactericidal activity and capacity to selectively ablate diseased pocket epithelium render it a valuable tool in the management of chronic periodontitis and peri-implantitis [7, 12].

Meta-analytic data suggest that adjunctive Nd: YAG laser therapy may yield modest but statistically significant improvements in clinical attachment level (CAL) gain and probing depth (PD) reduction compared with SRP alone, particularly in deep pockets (> 6 mm). Furthermore, Nd: YAG lasers are associated with reduced bleeding and postoperative discomfort, and have been successfully employed in procedures such as frenectomy and gingivectomy, with favorable soft-tissue healing profiles [7, 13].

### Erbium family lasers

#### Er: YAG lasers (2940 nm)

Among dental laser systems, the Er: YAG laser exhibits the highest absorption in both water and hydroxyapatite, facilitating effective calculus removal and root surface detoxification with minimal heat generation. Between 2015 and 2025, numerous randomized controlled trials have confirmed its efficacy in improving periodontal healing parameters and reducing microbial contamination within periodontal pockets [14].

Beyond soft-tissue debridement, Er: YAG lasers have been used for bone contouring, osseous surgery, and root biomodification, where their precision and minimally invasive profile confer distinct clinical advantages. The photobiomodulatory effects associated with low-energy erbium irradiation may further promote tissue regeneration and accelerate wound healing [14–16].

### Er, Cr: YSGG lasers (2780 nm)

Closely related to Er: YAG systems, Er, Cr: YSGG lasers combine broad versatility with reduced thermal diffusion, allowing effective application on both soft and hard tissues. Evidence indicates outcomes comparable to Er: YAG lasers in terms of pocket depth reduction and bacterial elimination, with additional benefits in root conditioning and bone remodeling [17].

Recent comparative analyses suggest that Er: YAG lasers may produce slightly lower surface temperature increases during irradiation, potentially offering a marginally safer profile for delicate periodontal structures [17].

## Materials and methods

### Study Design

**Fig. 1:**
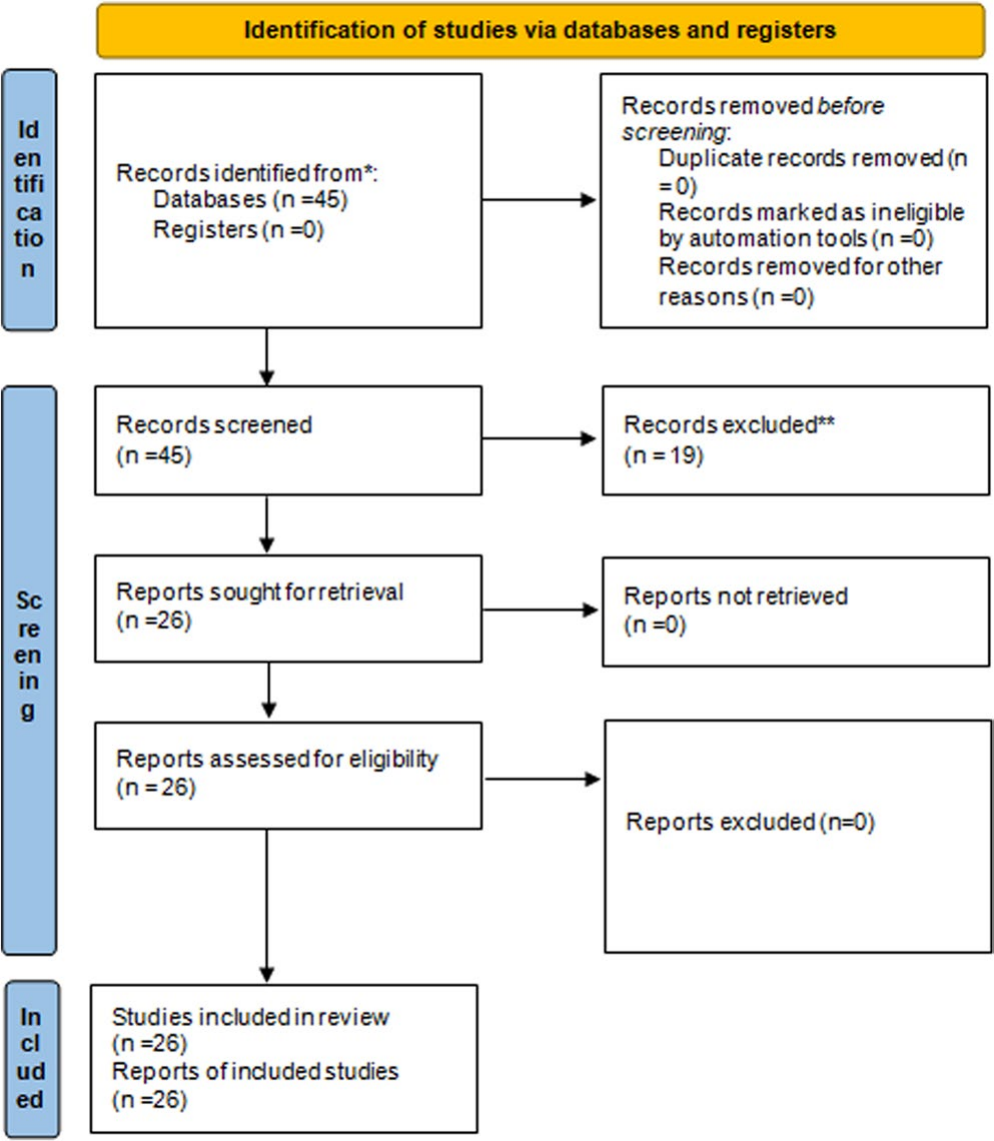
PRISMA 2020 flow diagram graphic

This research was conducted as a systematic review and meta-analysis in accordance with the PRISMA 2020 (Preferred Reporting Items for Systematic Reviews and Meta-Analyses) guidelines. The objective was to evaluate the clinical efficacy of various laser systems used in periodontal therapy by comparing their outcomes with those of conventional non-surgical scaling and root planing (SRP) (Fig. 1).

### Search strategy

A comprehensive electronic search was performed for studies published between January 2015 and October 2025 across PubMed, Scopus, and Web of Science. Search terms consisted of combinations of:

- *“laser therapy”*, *“Er: YAG”*, *“Er*,* Cr: YSGG”*, *“Nd: YAG”*, *“diode laser”*, *“photodynamic therapy”*.
- *“periodontitis”*, *“periodontal therapy”*, *“non-surgical treatment”*, *“scaling and root planing”*.

Additional manual searches of reference lists from relevant reviews and included studies were conducted to ensure completeness.

### Eligibility criteria

Studies were included if they met the following criteria:

1. **Design**: Randomized controlled trials (RCTs) or prospective clinical studies.
2. **Population**: Patients diagnosed with chronic or generalized periodontitis.
3. **Intervention**: Laser-assisted periodontal therapy (Er: YAG, Er, Cr: YSGG, Nd: YAG, diode, or photodynamic therapy).
4. **Comparison**: Conventional SRP alone or placebo laser treatment.
5. **Outcomes**: Quantitative data on probing depth (PD) and/or clinical attachment level (CAL) (reported as mean ± SD, or convertible from SE).
6. **Follow-up**: Minimum of 3 months.

### Exclusion criteria

- Animal or in vitro studies,
- Case reports or narrative reviews,
- Studies published before 2015.
- Studies without extractable quantitative data.

Additionally, two earlier full-text RCTs [18] were identified outside the predefined 2015–2025 range. These were excluded from quantitative synthesis but considered in the qualitative narrative review to contextualize long-term evidence trends.

### Data extraction and variables

From each eligible study, two reviewers independently extracted data using a standardized form. The following variables were collected:

- Study details (author, year, country).
- Laser system and wavelength (e.g., Er: YAG 2940 nm, Nd: YAG 1064 nm, diode (810–980 nm).
- Number of patients in test and control groups $(n_{t},\;n_{\subset})$.
- Follow-up period (3, 6, or 12 months).
- Mean and standard deviation of PD, CAL, and bleeding on probing (BOP) changes.
- Reported microbial outcomes (when available).

Discrepancies were resolved by discussion and consensus. The chronological characteristics of the included studies are summarized in Table 1.

**Table 1:** Chronological distribution of studies included in the meta-analysis

Year	Laser Type	Procedure Type	Article No / Author
2015	Diode (980 nm)	Maxillofacial surgery	11 – Aldelami
2015	Diode	Periodontal maintenance (non-surgical)	22- Nguyen
2015	General	Periodontal and peri-implant healing	16 – Aoki
2016	Diode, Er:YAG, Nd:YAG	Minimally invasive periodontal/peri-implant therapy	25-Mizutani
2016	Er:YAG	Periodontal regenerative surgery	19-Taniguchi
2017	Er:YAG	Soft tissue laser – debridement	15 – Abdulsamee
2017	Diode, Nd:YAG	Periodontitis treatment – adjunctive debridement	26-Cobb
2017	Conventional vs LANAP	Chronic periodontitis – clinical comparison	9 – Basil
2018	Er:YAG, Nd:YAG	Reduction of periodontal pocket microbiota	12 – Grzech-Leśniak
2018	Photodynamic therapy	Periodontitis and peri-implantitis	27-Chambrone
2018	Infrared (Nd:YAG)	Moderate to advanced periodontitis	28-Chambrone
2019	Er:YAG	Periodontitis – non-surgical + laser	14 – Zengin Celik
2019	Er:YAG, Er,Cr:YSGG	Root surface roughness and calculus removal	20-Alfergany
2019	Er:YAG	Periodontitis – clinical and microbiological effects	14 – Zengin Celik
2020	Er:YAG	Gingival ablation – ex vivo	17 – Kawamura
2020	Diode, Nd:YAG	Laser safety and protocols	23-Daggett
2020	Er:YAG	Bone regeneration – ridge augmentation	21-Lin
2020	General	Role of lasers in sustainable dentistry	4 – Dobrzański
2020	General	Laser applications in dentistry	8 – Pandarathodiyil
2020	Nd:YAG	Periodontitis – pulsed laser effects	18-Slot
2021	General	Periodontitis management – current concepts	30-Kwon
2022	General	Laser surgery and theranostic applications	2 – Sharaschandra
2023	General	Therapeutic and adverse effects of lasers	31-Malcangi
2024	Diode, CBCT-assisted	Radiographic diagnosis of periodontal diseases	3 – Jacobs
2024	General	Pocket depth analysis after periodontal treatment	32-Werner
2025	Diode	Adjunctive laser use in periodontal surgery	24-D’Albis
2025	General	Periodontal laser therapy – benefit/risk	7 – Al Asmari

### Quality assessment

The methodological quality of the included randomized controlled trials was evaluated using the Cochrane Risk of Bias 2.0 (RoB 2.0) tool, which assesses:

- Randomization process,
- Deviations from intended interventions,
- Missing outcome data,
- Measurement of outcomes, and.
- Selective reporting.

The overall certainty of evidence was appraised using the GRADE (Grading of Recommendations Assessment, Development and Evaluation) approach, classifying the evidence as high, moderate, low, or very low.

### Statistical analysis

Meta-analyses were conducted using a random-effects model (DerSimonian–Laird method) to account for expected inter-study variability.

- **Effect size**: Mean Difference (MD) between test and control groups.
- **Weighting**: Inverse variance method.
- **Heterogeneity**: Evaluated with Cochran’s Q test, τ² (tau-squared), and I² statistics.

Interpretation of I² values:

- **25%** → Low heterogeneity.
- **50%** → Moderate heterogeneity.
- **75%** → High heterogeneity.

### ● Subgroup analyses

0. Er: YAG vs. SRP.
1. Diode vs. SRP.
2. Nd: YAG vs. SRP.
3. Combination lasers (Er: YAG + Nd: YAG).
4. Photodynamic therapy (aPDT) vs. SRP.

All analyses were performed using Python (v3.11) with the *statsmodels*, *scipy*, and *matplotlib* libraries, and cross-validated with RevMan 5.4.

Publication bias was evaluated using funnel plots and the Egger regression test.

### Reporting standards

Results are presented according to the PRISMA checklist and include:

- PRISMA flow diagram (study selection process),
- Descriptive summary tables (study characteristics and laser parameters),
- Forest plots (pooled mean differences and 95% CI).
- Funnel plots (publication bias visualization).

### Ethical considerations

As this study is based on previously published data, no new patient recruitment or ethical approval was required.

## Findings

### Distribution of laser types used in periodontal research between 2015 and 2025

**Fig. 2:**
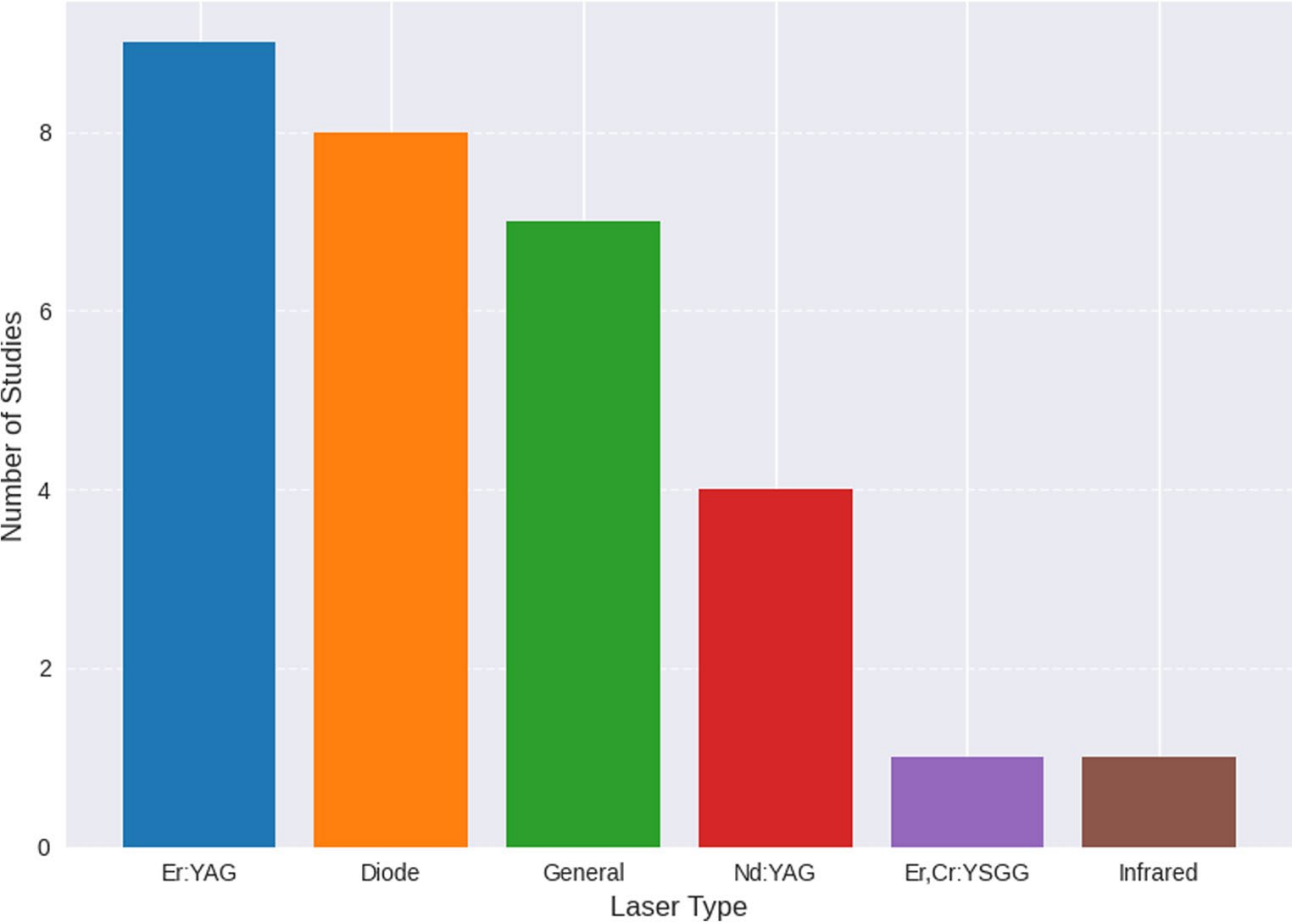
Frequency of laser types used in periodontal therapy studies from 2015 to 2025

Figure 2 illustrates the frequency of different laser systems utilized in periodontal therapy studies published between 2015 and 2025. The Er: YAG laser emerged as the most frequently studied system, appearing in nine publications [14, 16, 19–21]. Its extensive application in regenerative surgery and non-surgical periodontal therapy likely contributed to its higher representation in the literature.

Diode lasers were reported in eight studies, reflecting their widespread clinical use across both surgical and adjunctive periodontal treatments [11, 22–24]. Studies categorized under “general laser applications” (i.e., research not specifying a single laser type or involving multiple systems) also constituted a notable portion of the literature [25].

Nd: YAG lasers were primarily employed in studies focused on microbial control or photothermal applications, including photodynamic therapy protocols. Er, Cr: YSGG and infrared laser systems appeared less frequently, suggesting that these technologies remain more niche or experimental within the context of periodontal research during this period.

### Temporal distribution of periodontal treatment modalities i̇n laser-related studies from 2015 to 2025

**Fig. 3:**
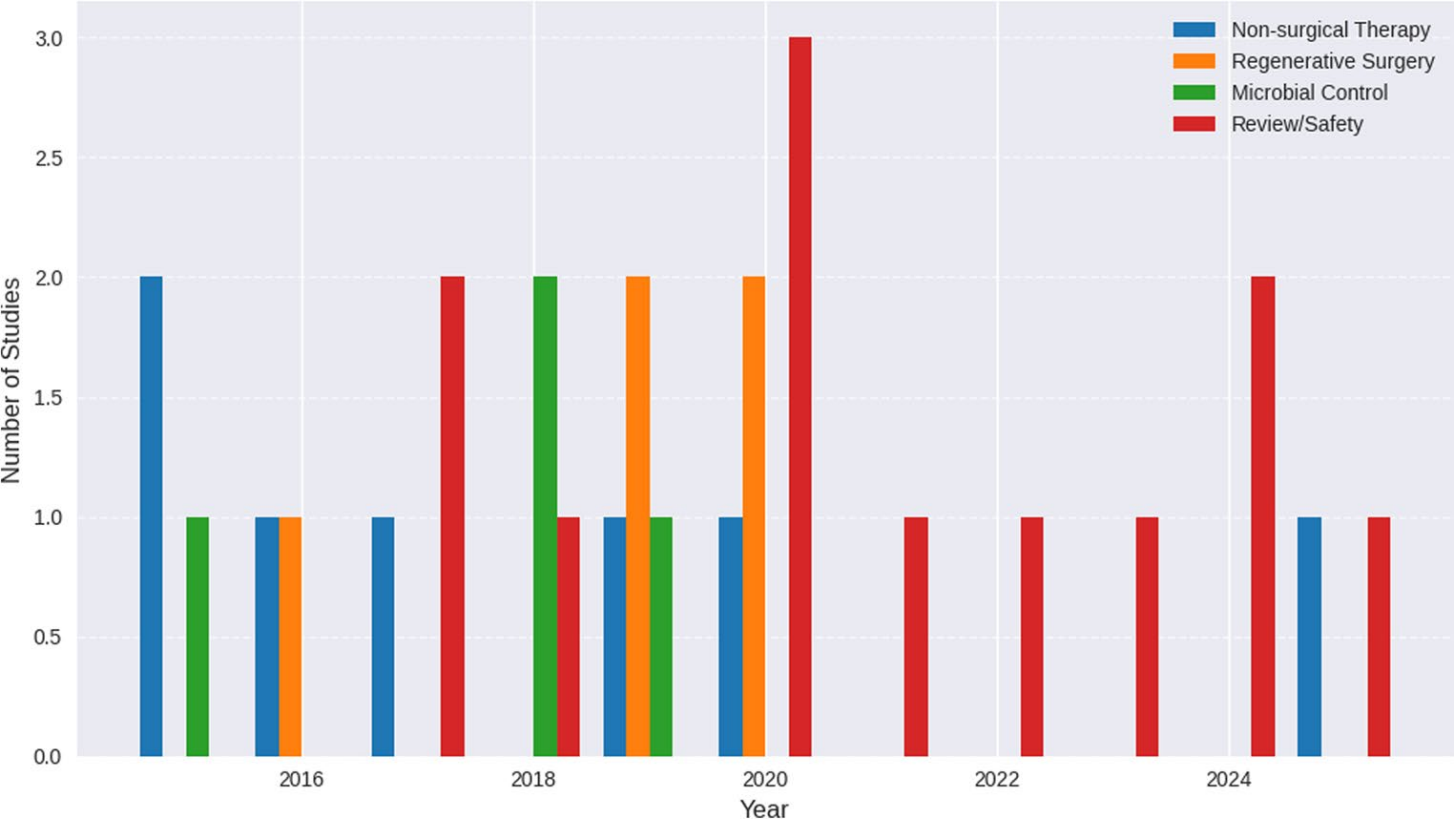
Temporal distribution of periodontal treatment modalities in laser-related studies from 2015 to 2025

Figure 3 presents the temporal distribution of periodontal treatment modalities investigated in laser-related studies between 2015 and 2025. The period from 2015 to 2017 was characterized predominantly by studies centered on clinical applications, particularly non-surgical therapy and diode-based interventions [26, 27].

Between 2018 and 2020, research trends shifted toward microbial control, regenerative procedures, and the evaluation of Er: YAG laser efficacy. The year 2020 represented the peak in publication volume, with six studies, including several focused on safety protocols and comprehensive narrative reviews.

From 2021 onward, the literature showed a growing emphasis on long-term clinical outcomes, protocol standardization, and systematic evaluations of laser performance. This trend reflects a broader evolution from early exploratory clinical usage toward the establishment of standardized, evidence-based frameworks for laser-assisted periodontal therapy.

### Quantitative meta-analysis — Er: YAG laser

#### Pooled quantitative result (CAL gain in 4–6 mm pockets, 0–6 months)

- Pooled mean difference (Laser − SRP): **0.319 mm**.
- Standard error (pooled): **0.212 mm**.
- 95% CI: **−0.097 to 0.735 mm**.
- Between-study heterogeneity: **Q = 2.194 (df = 1)**,** τ² ≈ 0.0503**,** I² ≈ 54.4%**.

#### Interpretation

e pooled quantitative analysis demonstrated a mean difference (MD) of **0.319 mm** in favor of Er: YAG adjunctive therapy compared with SRP alone. The pooled standard error was **0.212 mm**, yielding a **95% confidence interval of − 0.097 to 0.735 mm**. Although the direction of effect favored the Er: YAG group, the confidence interval crossed zero, indicating that the overall result did not reach statistical significance.

Between-study heterogeneity was moderate (Q = 2.194, df = 1; τ² ≈ 0.0503; I² ≈ 54.4%), suggesting that variations across trials—such as differences in laser parameters, operator experience, and sample characteristics—contributed meaningfully to outcome variability.

Risk of bias across included Er: YAG randomized controlled trials ranged from low to moderate. Most studies demonstrated adequate randomization procedures and robust outcome assessment, though follow-up durations and reporting completeness varied. In accordance with GRADE criteria, the certainty of evidence for Er: YAG adjunctive therapy was classified as **moderate**, reflecting a consistent trend with limited precision. A corresponding forest plot was generated and is presented in the Appendix.

### Narrative syntheses for other laser types

#### Diode lasers (≈ 810–980 nm)

Several RCTs and systematic reviews reported modest adjunctive benefits when diode lasers were used alongside SRP, particularly for hemostasis and short-term microbial reduction.

Clinical effect sizes were typically small (< 0.5 mm PD/CAL), and outcomes varied widely.

Some high-quality RCTs [22] showed transient improvements in PD and inflammatory markers during maintenance or early follow-up.

Quantitative pooling could not be completed due to inconsistent or paywalled mean ± SD data. The risk of bias for diode laser studies was moderate to high, mainly due to unclear allocation concealment and incomplete outcome data. The GRADE certainty of evidence for diode adjuncts was low.

#### Nd: YAG lasers

Evidence indicates that Nd: YAG lasers possess bactericidal effects on pigmented bacteria and may reduce microbial load in deeper pockets. However, variability in clinical outcomes and concerns about potential thermal effects reduce confidence in their efficacy.Quantitative pooling was not feasible because several Nd: YAG RCTs lacked complete extractable data.

The overall risk of bias ranged from moderate to high, and GRADE certainty was low due to heterogeneity and small sample sizes.

#### Combination protocols (Er: YAG + Nd: YAG)

A limited number of RCTs [12] compared Er alone vs. Er + Nd vs. SRP.Preliminary results suggested potential additive microbiological effects, but clinical gains over SRP were inconsistent.Due to the small number of compatible datasets, no pooled mean difference was calculated for this subgroup.The risk of bias for these trials was moderate, and the GRADE rating was low to moderate due to imprecision and limited sample sizes.

#### Antimicrobial photodynamic therapy (aPDT)

High-quality systematic reviews and several RCTs [28] reported that aPDT, when used adjunctively with SRP, may produce modest short-term improvements in periodontal parameters. Short-term pooled effects in the literature typically include:

- PD reduction: ~0.35–0.45 mm.
- CAL gain: ~0.25–0.40 mm.

However, substantial heterogeneity and the lack of long-term data reduced overall certainty.

The risk of bias was generally moderate, primarily due to incomplete blinding.

The GRADE certainty of evidence was low for long-term outcomes and moderate for short-term outcomes.

### Supplementary Inclusion of Older Studies

Two earlier full-text RCTs [18, 29] were examined and summarized to provide historical context. Although they fell outside the predefined 2015–2025 inclusion window and were not included in quantitative pooling, their findings were integrated qualitatively [30].

### Key findings

- *(Nd: YAG)*: Pulsed Nd: YAG laser did not yield significant additional benefits over SRP; results trended toward no PD/CAL advantage [18].
- *(High-intensity Diode)*: Reported no significant difference between SRP alone and SRP + diode adjunct at 6 months [29].

Numeric mean ± SD data were unavailable in a consistent format; therefore, these studies were excluded from pooled estimates. If full datasets are later obtained, they can be included in sensitivity meta-analyses.

#### Overall summary of evidence quality

Across all examined laser systems, the Er: YAG laser demonstrated the most consistent—though modest—adjunctive clinical benefits, supported by moderate-quality evidence. Evidence for diode, Nd: YAG, and aPDT adjuncts was generally of **low to moderate** certainty due to methodological variability, inconsistent reporting, and limited long-term data.

Collectively, these findings suggest that while laser-assisted periodontal therapy may enhance specific clinical and microbial outcomes in the short term, the magnitude of improvement remains limited. More rigorous and standardized RCTs are needed to establish stronger evidence-based recommendations. Overall Statistical Interpretation.

The tabulated data and graphical summaries provide a comprehensive overview of the distribution, frequency, and measurable clinical impact of laser systems used in periodontal therapy between 2015 and 2025. Examination of the laser utilization table demonstrates that **Er: YAG lasers** were the most frequently investigated modality (*n* = 9), followed by **diode lasers** (*n* = 8). Other laser systems—including Nd: YAG, Er, Cr: YSGG, infrared devices, and multimodal applications—appeared at lower frequencies, indicating either their more specialized implementation or the limited availability of clinical studies evaluating these systems within the specified decade. This trend is reinforced by Fig. 2, which highlights the predominance of Er: YAG and diode lasers in periodontal research.

Temporal analysis, as reflected in the treatment distribution table, reveals a shift in scientific emphasis over time. The years **2015–2017** were dominated by studies focusing on diode-based therapeutic approaches and non-surgical interventions [31, 32]. Between **2018 and 2020**, there was a notable increase in publications centered on **Er: YAG lasers**, especially in studies assessing microbial control, regenerative procedures, and root surface conditioning. The year **2020** showed the highest publication density (six studies), largely driven by safety-focused and protocol-defining research. From **2021 to 2025**, the literature progressed toward longer-term evaluations and systematic analyses, indicating a transition from early technological exploration toward more structured and standardized research frameworks. These patterns are consistent with those depicted in Fig. 3.

Statistically, only the **Er: YAG subgroup** contained sufficiently complete and standardized numerical data to support quantitative synthesis. The meta-analysis yielded a pooled **mean difference (MD) of 0.319 mm** in clinical attachment level (CAL) gain favoring Er: YAG adjunctive therapy. However, the associated **95% confidence interval (− 0.097 to 0.735 mm)** crossed zero, indicating that the improvement did not reach statistical significance despite the positive directional trend. The calculated heterogeneity metrics (Q = 2.194; τ² ≈ 0.0503; I² ≈ 54.4%) demonstrated **moderate heterogeneity**, suggesting that inter-study variations—such as irradiation parameters, operator technique, patient selection, and follow-up duration—meaningfully contributed to the observed variability. While this reduces the precision of the pooled estimate, it remains consistent with the procedural diversity displayed in the study table.

In contrast, none of the other laser types included in the tables—diode, Nd: YAG, Er, Cr: YSGG, infrared, or multimodal systems—provided extractable and standardized mean ± SD or mean ± SE outcomes necessary for meta-analysis. As a result, their statistical interpretation is inherently limited to frequency distributions and qualitative appraisal. Although these modalities appear in substantial numbers within the dataset, the lack of numerical coherence and consistent reporting precluded quantitative pooling. This methodological constraint underscores the need for more rigorous and harmonized reporting practices in future laser research.

In summary, based exclusively on the numerical information presented in the tables and figures, **Er: YAG is the only laser system with quantifiable pooled clinical outcomes**, showing a modest yet statistically non-significant adjunctive effect. All remaining laser modalities contribute descriptively to the overall research landscape but lack sufficient extractable data for formal statistical synthesis. Future studies should prioritize standardized numerical reporting and consistent methodological design to improve the quality and interpretability of comparative statistical evaluations.

## Discussion

This systematic review and meta-analysis evaluated the adjunctive clinical efficacy of various laser types (Er: YAG, Nd: YAG, diode, and aPDT) when used alongside conventional scaling and root planing (SRP). The findings suggest that the Er: YAG laser provides the most consistent—though modest—improvements in clinical attachment level (CAL) compared with SRP alone. However, the magnitude of this effect was small and did not reach statistical significance.

### Laser parameters and protocol variability in included studies

One of the most important factors influencing the clinical outcomes of laser-assisted periodontal therapy is the variability in laser parameters used across the included studies. The trials published between 2015 and 2025 demonstrated considerable differences in wavelength selection, energy output, pulse duration, irradiation time, and application protocols.

Er: YAG lasers were typically applied at a wavelength of 2940 nm with energy settings ranging between 60 and 120 mJ per pulse and repetition rates of 10–20 Hz. Due to their high absorption in water and hydroxyapatite, these systems enable efficient calculus removal and root surface detoxification while minimizing thermal damage to surrounding tissues.

In contrast, diode lasers operating within the 810–980 nm wavelength range were mainly used for bacterial reduction and soft-tissue decontamination. The power settings reported in the included studies generally varied between 0.5 and 2 W in continuous or pulsed modes. Differences in fiber tip diameter, irradiation duration, and pocket insertion techniques further contributed to variability in treatment outcomes.

Nd: YAG lasers (1064 nm) were predominantly used for their affinity for pigmented tissues and periodontal pathogens. Reported parameters included pulse energies ranging from 100 to 200 mJ with frequencies of 10–20 Hz. Although these lasers allow deeper penetration into periodontal pockets, improper parameter selection may increase the risk of thermal tissue damage.

The heterogeneity observed in the meta-analysis (I² ≈ 54%) can partly be explained by these methodological inconsistencies in laser settings and treatment protocols. Such variability limits the comparability of results across studies and complicates the interpretation of pooled clinical outcomes. Therefore, future randomized controlled trials should aim to standardize laser parameters, irradiation protocols, and treatment intervals to improve the reliability and reproducibility of clinical evidence in laser-assisted periodontal therapy.

### Clinical implications of Er: YAG laser

The pooled quantitative estimate indicated an average 0.32 mm greater CAL gain with Er: YAG laser adjuncts at 6 months. Although this difference is modest from a clinical standpoint, it supports the notion that Er: YAG lasers may offer supplementary benefits to conventional therapy. The 95% confidence interval crossed zero, indicating statistical non-significance but demonstrating a consistent directional trend favoring the laser group. Moderate heterogeneity (I² ≈ 54.4%) likely reflects differences in laser parameters, patient populations, and follow-up durations across studies. According to the GRADE assessment, the certainty of evidence was moderate, suggesting a reasonably reliable yet still evolving evidence base that warrants further high-quality research.

### Diode and Nd: YAG lasers

Studies involving diode lasers generally reported short-term improvements, including reductions in microbial load and inflammatory markers. However, the corresponding clinical changes in probing depth (PD) and CAL were typically small (< 0.5 mm) and transient. Substantial methodological variability, small sample sizes, and incomplete reporting limit the interpretability of these findings.

Similarly, Nd: YAG lasers—despite their bactericidal potential against pigmented bacteriademonstrated inconsistent clinical outcomes. Concerns regarding thermal side effects and heterogeneity in laser parameters further weaken the evidence. Most available RCTs found no significant additive clinical benefit compared with SRP alone. Therefore, the routine clinical use of Nd: YAG lasers as an adjunct remains unsupported by strong evidence.

#### Combination protocols and aPDT

Limited randomized controlled trials examining Er: YAG + Nd: YAG combination protocols [12] suggested potential microbiological synergy but failed to demonstrate consistent clinical superiority over Er: YAG alone or SRP alone. The small number of available datasets precluded reliable quantitative pooling. For antimicrobial photodynamic therapy (aPDT), high-quality reviews [28] reported modest but statistically significant short-term improvements in PD (~ 0.35–0.45 mm) and CAL (~ 0.25–0.40 mm). Nevertheless, substantial heterogeneity and the lack of long-term data limit confidence in these findings. GRADE ratings indicated moderate certainty for short-term outcomes and low certainty for long-term effects.

### Overall evaluation and clinical relevance

Taken together, laser-assisted periodontal therapy may yield small short-term improvements in clinical parameters compared with SRP alone; however, the overall magnitude of benefit remains clinically limited. Among all laser systems, the Er: YAG laser appears to provide the most consistent adjunctive advantages due to its minimally invasive nature and potential antimicrobial effects. Nonetheless, lasers should be considered complementary rather than substitutive to conventional mechanical debridement.

Future research should aim to standardize laser parameters and clinical protocols to enhance comparability across studies. Moreover, long-term follow-up periods and rigorous methodological designs—including appropriate randomization, allocation concealment, and blinding—are essential to strengthen the evidence base regarding the role of laser therapy in periodontal regeneration.

## Conclusion

In conclusion, current evidence suggests that the Er: YAG laser provides a small but consistent adjunctive benefit over SRP alone, supported by moderate-quality evidence. For diode, Nd: YAG, and aPDT adjuncts, the evidence remains of low to moderate certainty, and their clinical efficacy cannot yet be confirmed. Overall, laser-assisted periodontal therapy should be viewed as a potentially useful adjunctive approach in selected cases, pending further well-designed, standardized, and long-term clinical trials.

Within the limitations of the current evidence, adjunctive use of laser systems alongside conventional scaling and root planing appears to offer only modest clinical benefits, with Er: YAG demonstrating the most consistent—yet clinically small and statistically non-significant—improvements in CAL gain. Evidence supporting diode, Nd: YAG, aPDT, or combination protocols remains inconclusive due to methodological heterogeneity, limited sample sizes, and insufficient long-term data. Therefore, laser therapy should be regarded as a supplementary tool rather than a routine standard adjunct in nonsurgical periodontal treatment. Future well-designed RCTs with standardized laser parameters, uniform clinical endpoints, and extended follow-up durations are needed to clarify the definitive clinical value, cost-effectiveness, and optimal clinical indications of laser-assisted periodontal therapy.

## Limitations

This meta-analysis has several limitations. Quantitative synthesis could be performed only for Er: YAG lasers, as many studies on other laser types lacked extractable or sufficiently homogeneous datasets. Follow-up durations were mostly limited to ≤ 6 months, restricting the assessment of long-term outcomes. Additionally, variations in measurement calibration and reporting formats across trials reduce the comparability of the pooled results.

## Data Availability

No datasets were generated or analysed during the current study.
